# Alveolar Macrophages Prevent Lethal Influenza Pneumonia By Inhibiting Infection Of Type-1 Alveolar Epithelial Cells

**DOI:** 10.1371/journal.ppat.1006140

**Published:** 2017-01-13

**Authors:** Amber Cardani, Adam Boulton, Taeg S. Kim, Thomas J. Braciale

**Affiliations:** 1 Beirne B. Carter Center for Immunology Research, University of Virginia, Charlottesville, Virginia, United States of America; 2 Department of Microbiology, University of Virginia, Charlottesville, Virginia, United States of America; 3 Department of Molecular Physiology and Biological Physics, University of Virginia, Charlottesville, Virginia, United States of America; 4 Department of Pathology, University of Virginia, Charlottesville, Virginia, United States of America; St. Jude Children's Research Hospital, UNITED STATES

## Abstract

The Influenza A virus (IAV) is a major human pathogen that produces significant morbidity and mortality. To explore the contribution of alveolar macrophages (AlvMΦs) in regulating the severity of IAV infection we employed a murine model in which the Core Binding Factor Beta gene is conditionally disrupted in myeloid cells. These mice exhibit a selective deficiency in AlvMΦs. Following IAV infection these AlvMΦ deficient mice developed severe diffuse alveolar damage, lethal respiratory compromise, and consequent lethality. Lethal injury in these mice resulted from increased infection of their Type-1 Alveolar Epithelial Cells (T1AECs) and the subsequent elimination of the infected T1AECs by the adaptive immune T cell response. Further analysis indicated AlvMΦ-mediated suppression of the cysteinyl leukotriene (cysLT) pathway genes in T1AECs *in vivo* and *in vitro*. Inhibition of the cysLT pathway enzymes in a T1AECs cell line reduced the susceptibility of T1AECs to IAV infection, suggesting that AlvMΦ-mediated suppression of this pathway contributes to the resistance of T1AECs to IAV infection. Furthermore, inhibition of the cysLT pathway enzymes, as well as blockade of the cysteinyl leukotriene receptors in the AlvMΦ deficient mice reduced the susceptibility of their T1AECs to IAV infection and protected these mice from lethal infection. These results suggest that AlvMΦs may utilize a previously unappreciated mechanism to protect T1AECs against IAV infection, and thereby reduce the severity of infection. The findings further suggest that the cysLT pathway and the receptors for cysLT metabolites represent potential therapeutic targets in severe IAV infection.

## Author Summary

A primary feature of lethal influenza infection is viral pneumonia. Influenza viral pneumonia is caused by the direct infection of alveolar epithelial cells, which subsequently causes extensive alveolar inflammation and injury. Clinically this pathology manifests as diffuse alveolar damage leading to acute respiratory distress syndrome. As alveolar macrophages are positioned in the alveoli, they are the ideally localized to be a first-line of defense against alveolar invading pathogens, such as influenza. To explore the contribution of alveolar macrophages to the development of lethal influenza pneumonia, we generated a novel mouse model with a selective deficiency in alveolar macrophages. As a result of the alveolar macrophage deficiency, these mice developed severe diffuse alveolar damage and lethal respiratory compromise after influenza infection. Lethal injury resulted from increased infection of type-1 alveolar epithelial cells, and the elimination of these infected cells by effector T cells. Further analysis indicated that in order to render type 1 cells resistant to influenza infection, alveolar macrophages suppress leukotrieneD4 production and autocrine-signaling in type 1 cells. These results suggest that alveolar macrophages play a previously unappreciated role in protecting type 1 alveolar epithelial cells against IAV infection, and thus the severity of infection.

## Introduction

The Influenza A virus (IAV) is a major human pathogen. In the United States alone, IAV infections are associated with more than 20,000 deaths and 300,000 hospitalizations annually [1]. IAV is a negative sense RNA virus that is transmitted by aerosol and fomites [2]. Upon inhalation, IAV primarily infects and replicates in respiratory epithelial cells. After completion of the replication cycle the newly formed virions are released apically from the infected cells back into the pulmonary airspaces, allowing the virions to reach the more distal cells of the respiratory tract as the infection evolves [3].

Eventually the infectious IAV virions reach the terminal airways, which are lined by type 1 and type 2 AECs (T1 and T2AEC). As T1AECs are responsible for gas exchange, extensive infection and the subsequent elimination of these cells can lead to severe pulmonary compromise [4,5]. In the murine model of IAV infection, one of the prominent differences between infection with highly pathogenic IAV strains and strains producing limited morbidity and mortality, is the degree to which T1AECs become infected and are subsequently eliminated [6,7]. Furthermore, diffuse alveolar damage (DAD), defined by the presence of fibrin deposition and alveolar hyaline membrane formation, as well as viral infection of alveolar epithelial cells are frequently found in autopsies of severe clinical IAV infection [4,8]. Taken together, these data strongly suggest that the degree of alveolar epithelium infection by IAV, and the extent of the resulting injury, is one the many crucial regulators of the outcome of IAV infection.

Recently, several reports have implicated the lung resident alveolar macrophages (AlvMΦs) as critical modulators of IAV disease severity and the development of lethal pulmonary injury [9–12]. However, the mechanism(s) by which AlvMΦs influence the outcome of IAV infection has yet to be clearly determined, although an inability to clear cellular debris and exudates has been implicated. AlvMΦs are classically thought to be negative immune regulators and thereby inhibit inflammatory responses to harmless inhaled antigens [13]. Conversely, their location within the terminal airways also suggests that AlvMΦs are one of the first innate immune cell types to encounter any inhaled potentially harmful microbes. Therefore, AlvMΦs are also believed to be an important initiator of the inflammatory response during bacterial infections, making them an important first line of defense against lower respiratory tract infection [14]. In the clinical setting, AlvMΦ dysfunction has been observed in multiple disease settings including asthma, allergies, chronic obstructive pulmonary disease, pulmonary fibrosis, smoking related lung disease, and, in the complete absence of AlvMΦs, the development of pulmonary alveolar proteinosis [15–19].

While screening mice that had genes linked to myeloid lineage development knocked out specifically in myeloid cells, we identified a mouse model in which there was a cellular deficiency selectively in the AlvMΦ compartment. In this mouse model the expression of Core Binding Factor Beta (CBFβ), which regulates the activity of the Runx family of transcription factors essential for myelopoiesis, is selectively knocked out in myeloid cells expressing Lysozyme M (LysM). This was accomplished by crossing CBFβ floxed mice with mice that express Cre recombinase driven off of the LysM promoter (CBFβ^ΔLysM^) [20, 21]. As a consequence of CBFβ gene disruption, these mice exhibit a deficiency in AlvMΦs without detectable consequences in the number or function of other LysM expressing cells in the lung and spleen, thereby allowing us to better define and elucidate the mechanisms by which AlvMΦs regulate the outcome of influenza infection.

We report here that the AlvMΦ deficient CBFβ^ΔLysM^ mice are highly susceptible to lethal IAV infection. With the exception of their markedly reduced number of AlvMΦs, CBFβ^ΔLysM^ mice exhibit no deficiency in innate or adaptive immune cells in the lungs prior to or following IAV infection. The AlvMΦ deficiency did result in a marked increase in susceptibility of T1AECs to IAV infection, resulting in the development of DAD and lethal injury following infection. Indeed, a precipitous decline in respiratory function and development of lethal injury in these AlvMΦ deficient mice was associated with the onset of the adaptive immune response in the infected lungs, and immune mediated elimination of the infected T1AECs.

We further demonstrate that the AlvMΦ-conferred T1AEC resistance to IAV infection was associated with the suppression of the genes for enzymes involved in the 5-lipoxygenase (5-LOX) to cysteinyl leukotriene (cysLT) pathway in T1AECs from infected lungs. Consistent with a role for AlvMΦs in regulating the susceptibility of T1AECs to IAV infection through control of the cysLT pathway, blockade/knockdown of cysLT pathway enzymes in T1AECs *in vitro* or antagonism of the cysLT pathway and the cysteinyl leukotriene receptor 1 *in vivo* reduced the susceptibility of T1AECs to IAV infection and rendered the AlvMΦ deficient CBFβ^ΔLysM^ mice resistant to lethal IAV infection.

## Results

### Characterization of the Conditional CBFβ Deficient Mice

**Fig 1:**
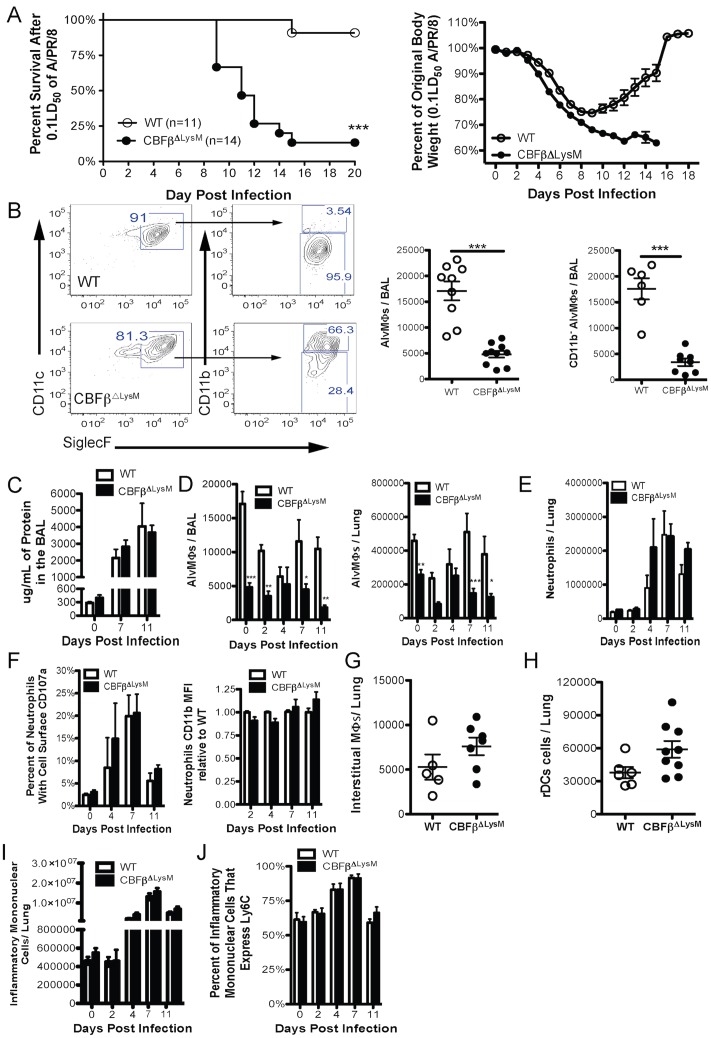
Alveolar macrophage deficient CBFβ^ΔLysM^ mice exhibit enhanced mortality after influenza infection. WT and CBFβ^ΔLysM^ mice were infected i.n. with a 0.1LD50 of A/PR/8. a) Survival (left) and weight loss (right) (with surviving CBFβ^ΔLysM^ mice removed) out to day 20 PI. b) Representative flow plots and total numbers of AlvMΦs (left) and CD11b^-^ AlvMΦs (right) in the BAL fluid at day 0 PI. c) Total protein detected in the BAL at the indicated days PI. d) Total number of AlvMΦs in the BAL and lungs at the indicated days PI. e) Total number of neutrophils in the lung and their f) percent with cell surface CD107a (first panel) and CD11b MFI (second panel) at the indicated days PI. g) Total numbers of lung interstitial macrophages and h) respiratory dendritic cells at day 0 PI. i) Total numbers of inflammatory mononuclear cells and j) percentage that are Ly6C^+^ in the lungs at the indicated days PI. Data were pooled from a minimum of 3 experiments with a total of 5–12 infected mice per genotype at each indicate time point. Error bars are standard error mean. Statistical analysis is a two- tailed non-paired students t test for single time points or 2-way ANOVA when multiple time points are present. * indicates P< .05, ** for P < .001 and *** for P < .001.

To assess the impact of disruption of the CBFβ gene in the myeloid lineage we examined the outcome of intranasal (i.n.) infection of CBFβ^ΔLysM^ mice and wild type (WT) control CBFβ^fl/fl^ littermates with a sublethal dose (0.1LD_50_) of the mouse adapted Influenza A strain A/PR/8 [H1N1]. As expected, infected WT mice survived and recovered from this inoculum dose (Fig 1a). However, CBFβ^ΔLysM^ mice exhibited markedly reduced survival (> 85% mortality) following infection (Fig 1a) suggesting that expression of CBFβ in one or more cell types of the myeloid lineage was critical for recovery from IAV infection.

Since several cell types of myeloid origin are affected by LysM driven Cre-mediated inactivation of the CBFβ gene, we employed the ROSA26 reporter mouse system, which allowed us to identify the cell type(s) responsive to LysM-Cre by Cre driven YFP expression. As Table 1 indicates, both before and after infection, efficient recombination (YFP expression) was primarily restricted to neutrophils and AlvMΦs, each of which displayed greater than 80% LysM-Cre driven recombination. By contrast, inflammatory mononuclear cells and respiratory dendritic cells were only modestly YFP^+^ (~ 20% or less) (Table 1) (Gating strategy S1 Fig).

**Table 1:** Pulmonary YFP expression in LysM-Cre x ROSA26 reporter mice prior to and during IAV infection.

Cell Type	Highest Rate of YFP Expression From Day 0-7PI
CD45-	0%
T cells	0%
NK cells	0%
Eos	0%
rDC	13%
AlvMΦs	85%
Neutrophils	84%
IMNCs	22%

The above reporter mouse analysis suggested that inactivation of the CBFβ gene by LysM-Cre would primarily affect the neutrophil and/or AlvMΦs lineages. However, published findings indicate that the RUNX TFs are essential early during neutrophil development, but are down regulated just as LysM expression is upregulated [22]. Therefore, we did not expect pulmonary neutrophil accumulation and function to be significantly impacted by the CBFβ deletion. By contrast, examination of the CD45^+^ cells in the bronchial alveolar lavage (BAL) fluid and lungs of naïve mice revealed markedly diminished numbers of AlvMΦs (CD45^+^, CD11c^+^, Siglec F^+^ cells) in the CBFβ^ΔLysM^ mice compared to their WT littermate controls (70%-80% reduction in the BAL and 50%-75% in the lung) (Fig 1b). In contrast to WT AlvMΦs, which are classically defined as CD11b^-^, the majority of the small number of AlvMΦs in the CBFβ^ΔLysM^ BAL fluid and lungs were CD11b^+^, however they still maintained typical macrophage morphology (Fig 1b and S2a Fig). Immature AlvMΦs are initially CD11b^+^, but down regulate CD11b as they mature/differentiate. Therefore, since CBFβ expression supports myeloid lineage development, the small number of CD11b^+^ AlvMΦs could represent cells at an early/intermediary stage in AlvMΦ development/ differentiation [23, 24]. Of note, the residual AlvMΦs in naive CBFβ^ΔLysM^ mice were sufficient to prevent the development of alveolar proteinosis as determined by BAL protein concentration and lung histology/morphology (Fig 1c and S2b Fig). After IAV infection there was a transient decrease in the number of AlvMΦs in the BAL of WT mice that began to recover by day 7 PI and progressively increased out to day 11 PI. In contrast, the AlvMΦ deficit in the CBFβ^ΔLysM^ mice became even more pronounced over time with few AlvMΦs (CD11b^-^ or CD11b^+^) detectable at day 7 PI and beyond (Fig 1d).

As expected, we observed no difference between WT and CBFβ^ΔLysM^ mice in their lung and BAL accumulation of neutrophils (CD45^+^, Siglec F^-^, CD11b^+^, Ly6G^+^ cells) before and during IAV infection (Fig 1e and S2d Fig). We also detected no difference in cell surface expression of CD107a (a marker of degranulation) or in the magnitude of CD11b expression (which is elevated on activated neutrophils) on pulmonary neutrophils following IAV infection in WT and CBFβ^ΔLysM^ mice (Fig 1f) [25]. In summary, these data suggest that, as expected, neutrophil infiltration and function during IAV infection is unaffected in CBFβ^ΔLysM^ mice.

There was also no deficit in the total number of pulmonary interstitial macrophages (CD45^+^, Siglec F^-^, CD11b^+^, F4/80^+^ cells) (Fig 1g) or respiratory dendritic cells (CD45^+^, CD11c^+^, MHCII^+^, Siglec F^-^, B220^-^ cells, either CD103^+^ or CD11b^+^) (Fig 1h) in WT and CBFβ^ΔLysM^ mice. LysM driven Cre recombination activity was also detected in a minor fraction of cells making up the heterogeneous population of inflammatory mononuclear cells (IMNCs) (Table 1). The absolute number of IMNCs (CD45^+^, CD11b^+^, Siglec F^-^, Ly6G^-^ cells) (Fig 1i and S2e Fig) and the frequency of IMNCs that express Ly6C (Fig 1j) before or during IAV infection were likewise unaffected in CBFβ^ΔLysM^ mice. As there was no difference in IMNC infiltration between wild type and CBFβ^ΔLysM^ mice, CCR2 expression by the mononuclear cells was not further evaluated. Interestingly, we also saw no difference in splenic macrophages, neutrophils, IMNCs and DCs in CBFβ^ΔLysM^ and WT mice (S2c Fig). These data suggest that the disruption of CBFβ in the CBFβ^ΔLysM^ mice had only a minimal, if any, effect on the development and effector response of these pulmonary mononuclear cell subsets and the innate immune response to IAV infection.

### Characterization of Virus Clearance and the Adaptive Immune Response in IAV Infected CBFβ^ΔLysM^ Mice

**Fig 2:**
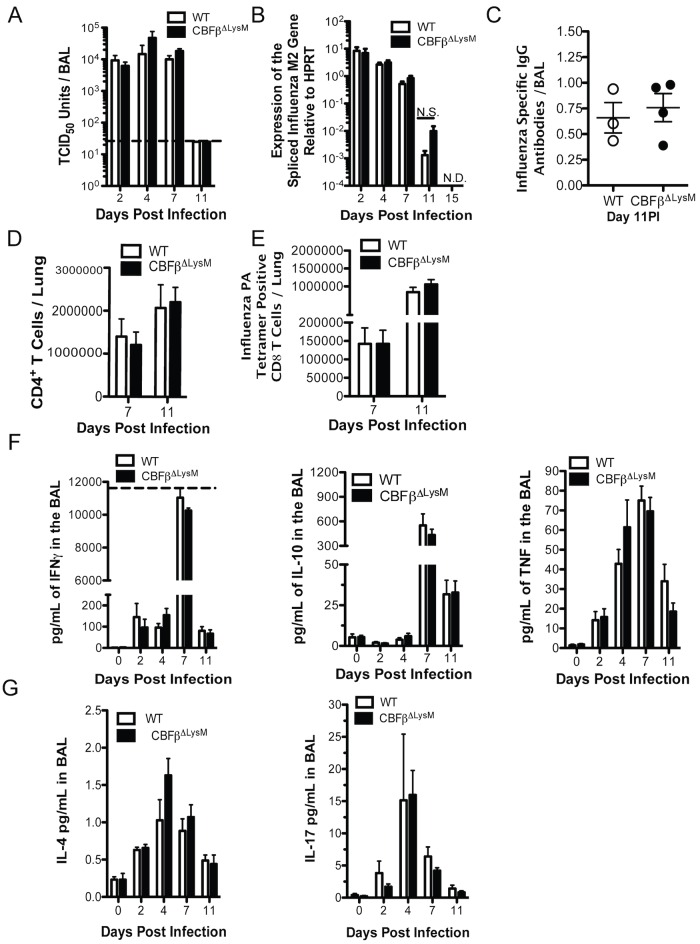
Virus clearance and adaptive immune responses in CBFβ^ΔLysM^ mice. WT and CBFβ^ΔLysM^ mice were infected i.n. with a 0.1LD50 dose of A/PR/8. Kinetics of virus replication and clearance as determined a) in BAL fluid by TCID50 units (dashed line is the lower limit of detection) and b) by qRT-PCR for the spliced IAV M2 gene at the indicated days PI. c) Representative levels of IAV specific IgG antibodies in the BAL fluid at day 11 PI. d) Total number of CD4 T cells and e) CD8 T cells positive for the IAV PA antigen tetramer in the lungs at the indicated days PI. Kinetics of BAL f)I FNγ, IL-10 and TNF g) IL-4 and IL-17. For IAV specific antibodies, data is representative of 2 experiments for a total of 5 WT and 6 CBFβ^ΔLysM^ mice. For all other data, data were pooled from a minimum of 3 experiments with a total of 4–9 mice per genotype at each indicate time point. Error bars are standard error mean. A 2-way ANOVA was used for statistical analysis. * indicates P< .05, ** for P < .001 and *** for P < .001. L.D. is limit of detection, NS is not significant, N.D. is not detectable.

In previous reports acute depletion of AlvMΦs prior to IAV infection was shown to result in enhanced virus titers and impaired adaptive T cell responses [9, 11, 12]. In order to determine if the loss of AlvMΦs in the CBFβ^ΔLysM^ mice resulted in uncontrolled virus replication, or had any impact on the IAV adaptive response, we next evaluated virus replication/clearance and the adaptive response in WT and CBFβ^ΔLysM^ mice. The CBFβ^ΔLysM^ mice succumbed to infection between days 8 and 15 PI (Fig 1a), which are following the characteristic onset of the adaptive immune response and concomitant virus clearance as observed in WT mice [26]. The tempo of IAV replication and clearance in the lungs was comparable for WT and CBFβ^ΔLysM^ mice as determined by BAL fluid virus titers (Fig 2a). It is noteworthy that infectious virus was no longer detectable by day 11 PI, when infected CBFβ^ΔLysM^ mice succumbed to infection. Infectious virus clearance was also confirmed by the analysis of viral gene expression in whole lung homogenates of WT and CBFβ^ΔLysM^ mice (Fig 2b), which was comparable, except for a statistically non-significant trend toward a slight delay in clearance of the spliced IAV M2 gene mRNA in CBFβ^ΔLysM^ mice (Fig 2b) [26].

The above findings indicate that virus clearance was normal in the CBFβ^ΔLysM^ mice, suggesting that the adaptive immune response to IAV was not affected. Indeed, following infection, BAL IAV-specific IgG antibodies (Fig 2c), total CD4 T cells (Fig 2d) and IAV-specific CD8 T cells (Fig 2e) in the CBFβ^ΔLysM^ mice were comparable to WT mice. Furthermore, kinetics of effector T cell derived cytokines IFNγ, IL-10, and TNF were similar between CBFβ^ΔLysM^ mice and their WT littermates (Fig 2f). Consistent with previously published observations we were unable to detect Th2 or Th17 T cell responses (Fig 2g) in the lungs of CBFβ^ΔLysM^ or WT mice. A multiplex analysis of 30 cytokines and chemokines (listed in the Methods section) in the BAL fluid at days 0, 2, 4, 7, and 11 PI also gave comparable values for WT and CBFβ^ΔLysM^ mice, except for a modest elevation in CCL2 (days 7 and 11 PI) and CXCL9 (day 11 PI) in infected CBFβ^ΔLysM^ mice. Cumulatively, these data indicate that the AlvMΦ deficiency has no demonstrable effect on the establishment of the anti-IAV adaptive immune responses and IAV clearance, or on the function and properties of the innate immune cells involved in the induction of adaptive immune responses, particularly rDCs.

### IAV Infection in AlvMΦ Deficient Mice Results in Severe Respiratory Insufficiency and Marked Diffuse Alveolar Damage

**Fig 3:**
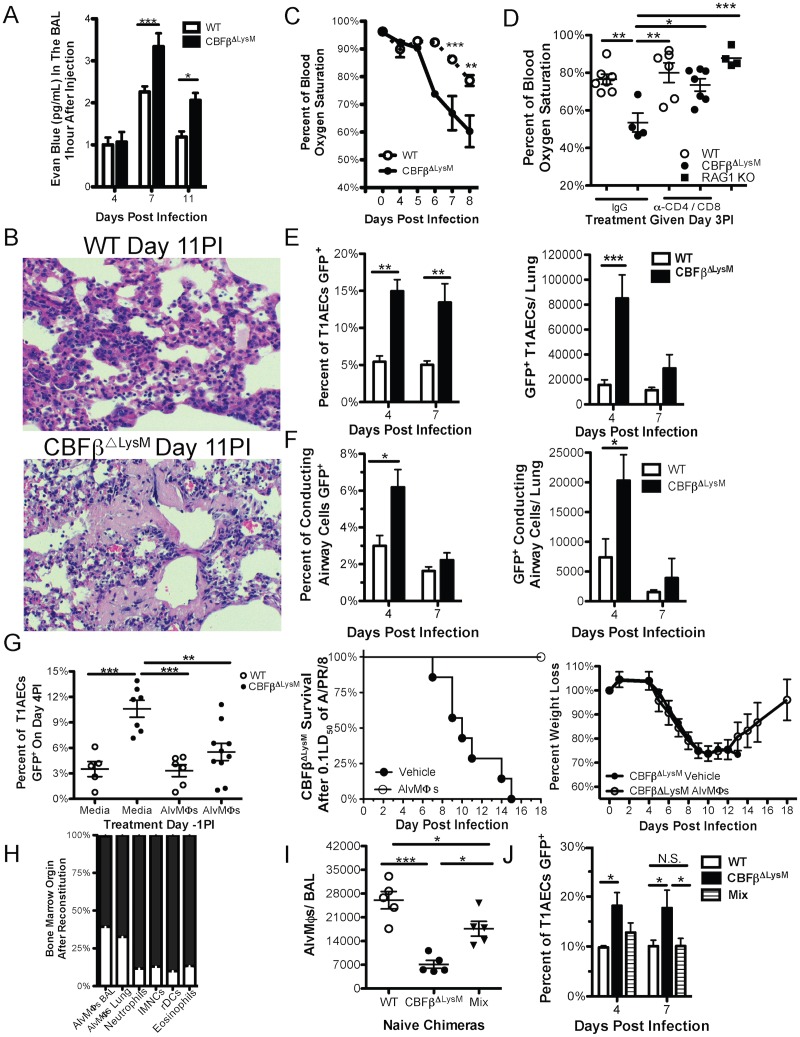
AlvMΦs regulate the susceptibility of type 1 alveolar epithelial cells to IAV infection. WT and CBFβ^ΔLysM^ mice were infected i.n. with a 0.1LD50 dose of a-c) 0.1LD50 of A/PR/8 or d-g) NS1-GFP A/PR/8. a) At the indicated days PI Evans Blue dye leak into the airspace was quantified. b) Representative H&E section images from day 12 PI IAV infected WT and CBFβ^ΔLysM^. c) Percent blood oxygen saturation at the indicated days PI. d) Day 7 PI blood oxygen saturation after i.p. injection of CD4 and CD8 depleting antibodies at day 3 PI. Percent of (left) and total numbers of (right) infected e) T1AECs and f) conducting airway epithelial cells at day 4 & 7 PI. g) AlvMΦs were transferred i.n. at day -1PI and (left) the percent of T1AECs that were infected at day 4PI was determined, as well as, (right) CBFβ^ΔLysM^ survival out to day 20PI (n = 4 CBFβ^ΔLysM^ mice treated with AlvMΦs). h-j) WT mice were irradiated and given either congenic CD45.1 WT bone marrow, CD45.2 CBFβ^ΔLysM^ bone marrow, or a mixture of 90% CBFβ^ΔLysM^ and 10% WT bone marrow (Mix). h) Seven weeks after reconstitution, the origin of the pulmonary myeloid cells in the mixed bone marrow chimera and i) the total number of AlvMΦs in the BAL was quantified. j) The percent of T1AECs that were infected (GFP^+^) at day 4 and 7 PI. Data were pooled from a minimum of 3 experiments with a total of 4–11 mice per genotype at each indicate time point. Error bars are standard error mean. For statistical analysis a two-tailed non-paired students t test, 1-way ANOVA or 2-way ANOVA was used where appropriate. * indicates P< .05, ** for P < .001 and *** for P < .001; NS is not significant.

The above results indicated that the quality and magnitude of the innate and adaptive host response, as well as the efficiency of virus clearance, in the IAV infected lungs were comparable between WT and CBFβ^ΔLysM^ mice. We did however observe a significant increase in erythrocyte extravasation into the airways/BAL fluid of CBFβ^ΔLysM^ mice when analyzed at day 7 and 11 PI. In order to directly quantify the extent of vascular leak in the infected lungs, we examined the accumulation of Evans Blue dye in the airways of WT and CBFβ^ΔLysM^ mice one hour after intravenous administration. While vascular leak was comparable in WT and CBFβ^ΔLysM^ mice on day 4 PI, by day 7 PI CBFβ^ΔLysM^ mice had markedly elevated Evans Blue dye accumulation in their airways (Fig 3a). These findings raised the possibility that there was substantial pulmonary capillary leak in the IAV infected CBFβ^ΔLysM^ mice, suggesting extensive alveolar damage was occurring at the time of onset of the adaptive immune response.

To assess whether the elevated vascular leak in the lungs of infected CBFβ^ΔLysM^ mice had any consequences on lung structure, we compared histopathologic changes in the lungs of infected WT and CBFβ^ΔLysM^ mice (Fig 3b). At day 12 PI both WT and CBFβ^ΔLysM^ mice displayed extensive interstitial inflammation and edema characteristic of severe IAV infection. However, the CBFβ^ΔLysM^ mice additionally had extensive intra-alveolar fibrin deposition, hyaline membrane formation and loss of alveolar wall integrity, reflecting histologic features characteristics of diffuse alveolar damage (Fig 3b). These findings suggest that the AlvMΦ deficiency in CBFβ^ΔLysM^ mice is linked to the development of enhanced alveolar injury following IAV infection.

A hallmark of enhanced alveolar injury with diffuse alveolar damage during severe IAV infection is compromised respiratory function, most notably diminished O_2_ exchange resulting in hypoxemia. When we analyzed blood O_2_ saturation in WT and CBFβ^ΔLysM^ mice following IAV infection, we observed that out to day 5 PI, O_2_ saturation was comparable in both WT and CBFβ^ΔLysM^ mice (Fig 3c). However at day 6 PI, corresponding with the onset of the IAV specific adaptive immune response in the lungs, there was a precipitous drop in O_2_ saturation in the CBFβ^ΔLysM^ mice, which resulted in progressive severe hypoxemia on subsequent days analyzed (Fig 3c).

It is noteworthy that there was only a modest initial decline in blood O_2_ saturation at day 4–5 PI in both WT and CBFβ^ΔLysM^ mice in spite of extensive IAV replication in the infected lungs at these early times (Fig 2a and 2b). The precipitous decline in O_2_ saturation coinciding with the onset of adaptive immune response raised the possibility that adaptive immune-mediate clearance of IAV infected epithelial cells was responsible for the rapid decline in O_2_ saturation CBFβ^ΔLysM^ mice. Indeed, the simultaneous depletion of CD4^+^ and CD8^+^ T cells at day 3 PI, thereby inhibiting the subsequent infiltration of these cells into the lungs, prevented the decline in O_2_ saturation at day 7 PI in CBFβ^ΔLysM^ mice (Fig 3d). By contrast, depletion of CD4^+^ and CD8^+^ T cells in infected WT mice had only a modest effect on O_2_ saturation (Fig 3d). Consistent with a role for the adaptive immune T cell response to IAV in the development of respiratory compromise, infected untreated adaptive immune deficient RAG KO mice also displayed only a modest decline in blood O_2_ saturation out to day 7 PI (Fig 3d).

### AlvMΦs Regulate the Susceptibility of Type 1 Alveolar Epithelial Cells to IAV Infection

The accumulated evidence demonstrating alveolar pulmonary vascular leak, histologic changes reflecting severe alveolar damage, and compromised pulmonary function (Fig 3a–3d) suggested that AECs were the likely target of the effector T cells in the CBFβ^ΔLysM^ mice. In order to determine if the T cell mediated alveolar injury in CBFβ^ΔLysM^ mice was related to the extent of AEC infection, we infected WT and CBFβ^ΔLysM^ mice with the reporter A/PR/8–NS1-GFP strain, which allows for the identification of infected cells by GFP expression [27]. Pulmonary epithelial cells were identified as CD45^-^, CD31^-^ and EpCAM^+^. Following published protocols, T1AECs were distinguished by podoplanin/T1α expression [28–31] and T2AECs were identified by cell surface MHCII expression, which we confirmed co-localized with intracellular pro-Surfactant Protein C staining in T2AECs from naïve lungs [31–35] (S3a and S3b Fig). CD45^-^, CD31^-^ and EpCAM^+^ cells that were negative for AEC lineage markers T1α or MHCII were grouped as bronchial/bronchiolar epithelial cells (referred to here as conducting airway epithelial cells) [35] (S3a and S3b Fig). (However, it should be noted that because of modest MHCII expression, the ratio of conducting airway cells to T2AECs might not be fully representative.) As Fig 3e demonstrates, the frequency of IAV infected T1AECs from CBFβ^ΔLysM^ mice was significantly elevated compared to the AlvMΦ sufficient WT mice at both day 4 & 7 PI. Importantly, while the frequency of infected T1AECs from the CBFβ^ΔLysM^ mice remained high at day 7 PI, the total number of infected T1AECs did decrease from day 4 to day 7 PI (Fig 3e), consistent with T cell mediated elimination of these virally infected cells at the latter time point.

In contrast, the susceptibility of the conducting airway cells and T2AECs from CBFβ^ΔLysM^ mice to IAV infection was increased at day 4 PI, but returned to WT levels by day 7 PI, before the death of the CBFβ^ΔLysM^ mice (Fig 3f and S3c Fig). Again, the total number of infected pulmonary epithelial cells decreased with the onset of the adaptive immune response in both AlvMΦ deficient CBFβ^ΔLysM^ mice and AlvMΦ sufficient WT mice. Given the three-fold increase in infection of T1AECs in CBFβ^ΔLysM^ mice, the two-fold increase in T2AEC infection by IAV was not unexpected (Fig 3e and S3c Fig). However, while both conducting and T2AEC infection rates returned to WT levels by day 7PI, the rate of T1AEC infection selectively remained high throughout infection in the AlvMΦ-deficient CBFβ^ΔLysM^ mice (Fig 3e and 3f and S3c Fig).

To determine if restoring AlvMΦs to CBFβ^ΔLysM^ mice would likewise restore resistance of T1AECs to IAV infection we employed two strategies. Firstly, we adoptively transferred 5x10^5^ WT AlvMΦs into CBFβ^ΔLysM^ mice by the i.n. route, one day prior to IAV infection. Transfer of AlvMΦs into CBFβ^ΔLysM^ mice rescued the resistance of their T1AECs to IAV infection, but had no impact on the susceptibility of T1AECs from WT mice (Fig 3g left panel). Importantly, this transfer of AlvMΦs into CBFβ^ΔLysM^ mice, which conferred resistance of T1AECs to infection, also rescued CBFβ^ΔLysM^ mice resistance to lethality after IAV infection (Fig 3g right panels).

Secondly, we constructed mixed bone marrow chimeric mice in which irradiated WT (CD45.2^+^) mice were reconstituted with either CD45.1^+^ WT bone marrow, CD45.2^+^ CBFβ^ΔLysM^ bone marrow, or a mixture of 90% CBFβ^ΔLysM^ and 10% WT bone marrow (Mix). Since the CBFβ^ΔLysM^ donor bone marrow would not be able to fully regenerate AlvMΦs in the irradiated recipients, a 10% WT bone marrow supplement was employed in the 90:10 mixed chimeras to selectively reconstitute the AlvMΦ compartment with WT cells, while all other bone marrow derived cell types would be primarily CBFβ^ΔLysM^ bone marrow derived.

Seven weeks after reconstitution, AlvMΦs in irradiated WT bone marrow reconstituted recipients were exclusively of donor bone marrow origin (S3d Fig). In the 90:10 mixed bone marrow recipients, the AlvMΦs were the only myeloid cells in the lungs that were derived from WT bone marrow at a frequency greater than 10% (Fig 3h), reflecting a partial restoration of AlvMΦ numbers (Fig 3i). Following IAV infection, the T1AECs from the mixed bone marrow chimeric mice with the partial AlvMΦs rescue demonstrated an enhanced resistance to IAV infection, which was comparable to the control irradiated WT bone marrow chimeras (Fig 3j).

Lastly, since neutrophils, like AlvMΦs, strongly express LysM (Table 1) we evaluated the impact of acute neutrophil depletion (by administration of the neutrophil depleting antibody IA8) on the susceptibility of T1AECs to infection. We observed no effect of neutrophil depletion on T1AEC susceptibility to IAV infection (S3e Fig). In sum these data further support the concept that CBFβ^ΔLysM^ mice have a selective quantitative deficiency in AlvMΦs, and that this deficit in AlvMΦs results in enhanced susceptibility of T1AECs cells to IAV infection.

### AlvMΦs Act Early in IAV Infection to Regulate the Susceptibility of T1AECs

**Fig 4:**
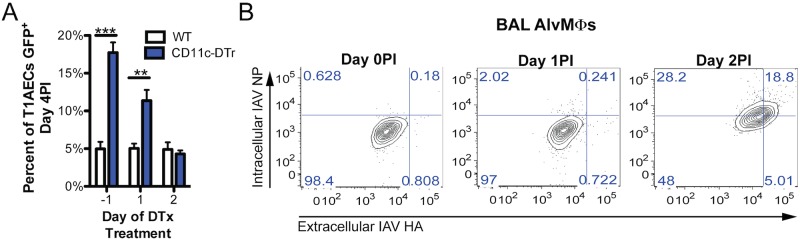
AlvMΦs function early in infection to confer resistance of type 1 alveolar epithelial cell to IAV infection. a) CD11c-DTr+ and WT control littermates were given 40ng of DTx i.n. at the indicated time points pre or post infection with NS1-GFP A/PR/8. The percentage of infected T1AECs was quantified at day 4PI. b) WT mice were infected i.n. with a 0.1LD50 of A/PR/8 and BAL AlvMΦs were isolated and stained for cell surface IAV HA antigen and intracellular IAV NP antigen at the indicated time points. Data were pooled from a minimum of 3 experiments with a total of 4–6 mice per genotype at each indicate time point. Flow plots are representative from 5 mice. Error bars are standard error mean. For statistical analysis 2-way ANOVA test were used. * indicates P< .05, ** for P < .001 and *** for P < .001.

Since, even in WT mice, AlvMΦ numbers are diminished by day 4 PI, (Fig 1d), we sought to determine when AlvMΦs conferred resistance of T1AECs to IAV infection. To do so, we employed a mouse model in which the diphtheria toxin (DTx) receptor is expressed under control of the CD11c promoter (CD11c-DTxR), allowing for the depletion of CD11c^+^ cells, including AlvMΦs, following DTx administration. DTx was administered prior to or up to 48hours following IAV infection. The susceptibility of T1AECs to IAV infection was evaluated at day 4 PI, prior to the onset of the adaptive immune response in the lungs, and therefore, before the impact of DTx administration on the elimination of rDCs would manifest. Similar to T1AEC from CBFβ^ΔLysM^ mice, T1AECs from CD11c-DTxR mice displayed enhanced susceptibility to IAV infection at day 4 PI when diphtheria toxin was administered i.n. on day -1 or day 1 PI (Fig 4a). On the other hand, when AlvMΦs were eliminated at day 2 PI, T1AEC susceptibility to IAV infection was comparable to T1AECs from DTx treated WT control mice (Fig 4a). These findings suggested that AlvMΦs function between days 1 and 2 PI to confer resistance of T1AECs to IAV infection. This is a time when there is minimal recruitment of other CD45^+^ cell types into the parenchyma or airways, and therefore, when AlvMΦs are the predominant CD45^+^ cell type in the airways.

To directly determine whether AlvMΦs were exposed to IAV at the time they were conferring resistance of T1AECs to infection, we evaluated the kinetics of AlvMΦ infection. As Fig 4b demonstrates, while viral genes were not detected in AlvMΦs isolated from the BAL prior to or on day 1 PI, cell surface HA and intracellular NP proteins were readily detectable by day 2 PI. This finding suggested that the regulation of T1AEC susceptibility by AlvMΦs was associated with AlvMΦ exposure to IAV.

### AlvMΦs Confer Resistance to IAV Infection by Regulating Expression of a Leukotriene Pathway in T1AEC

**Fig 5:**
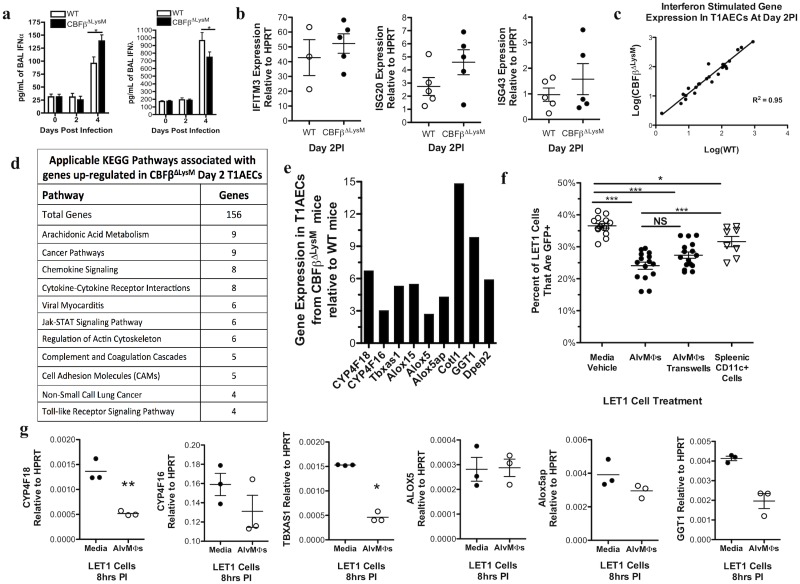
AlvMΦs suppress T1AEC expression of arachidonic acid metabolism pathway genes. a-e) WT and CBFβ^ΔLysM^ mice were infected i.n. with a 0.1LD_50_ of A/PR/8. a) IFNα and IFNλ protein in the BAL fluid prior to and during IAV infection. Representative interferon stimulated genes b) detected by qRT-PCR of whole lung homogenates c) and detected by RNAseq on sorted T1AECs at day 2 PI. d) Genes identified as over expressed in T1AECs from CBFβ^ΔLysM^ mice at day 2PI were grouped by pathway analysis. e) Arachidonic acid metabolism genes differentially expressed as determined by RNAseq. f) Percent of infected (GFP+) LET1 cells at 24hours post infection when cultured with media vehicle, AlvMΦs directly, AlvMΦs in transwell inserts or directly with splenic CD11c+ cells. g) Expression of the corresponding arachidonic acid metabolism pathway genes by qRT-PCR at 8 hours post infection in LET1 cells cultured alone or with AlvMΦs. a) BAL fluid was isolated from 4–10 mice per genotype at each indicate time point. c-d) 2–3 samples of pooled T1AECs from day 2 PI mice were used for RNAseq. For *in vitro* analyses, data were pooled from or is representative of a minimum of 3 experiments. Error bars are standard error mean. Statistical analysis is a) 2-way ANOVA, c) a linear regression analysis or f) 1-way ANOVA. * indicates P< .05, ** for P < .001 and *** for P < .001. N.S. is not significant.

Type I and III Interferons (IFNs) are important mediators of resistance to IAV infection and AlvMΦs have been implicated as one source of IFNs during infection [36]. Both Type I and Type III IFNs were detected at comparable levels in the BAL fluid of the AlvMΦ deficient CBFβ^ΔLysM^ and WT mice days 0–2 PI (Fig 5a). This is during the time frame in which AlvMΦs were demonstrated to confer resistance of T1AEC to IAV infection. Furthermore, there was no difference in the expression of representative IFN stimulated genes (ISGs) in whole lungs or sorted T1AECs isolated from CBFβ^ΔLysM^ and WT mice at day 2 PI (Fig 5b and 5c). Taken together, these findings suggested that, while IFNs are essential in controlling IAV infection, AlvMΦs were not utilizing IFNs to protect T1AEC from IAV infection.

To gain insight as to why T1AECs in the presence of AlvMΦs were more resistant to IAV infection, we carried out transcriptomic profiling (RNAseq) on T1AECs isolated from the lungs of the AlvMΦ deficient CBFβ^ΔLysM^ mice and AlvMΦ sufficient WT mice at day 2 PI, after AlvMΦ mediated resistance to infection had been conferred (Fig 4a).

RNAseq revealed that no genes were preferentially down regulated in the day 2 T1AECs from CBFβ^ΔLysM^ mice, but a number of genes were preferentially upregulated. Pathway analysis on the genes over expressed in the CBFβ^ΔLysM^ T1AECs using the NIH DAVID database revealed that nine of the genes were enzymes/co-factors involved in the arachidonic acid metabolism pathway (Fig 5d) [37, 38]. Four of the genes encode molecules involved in the cytochrome P450 (CYP4F18, CYP4F16), Thromboxane (Tbxas1) and 15-lipoxygenase (ALOX15) pathways of arachidonic acid metabolism (Fig 5e). The other five genes (ALOX5, ALOX5ap, Cotl1, GGT1 and DPEP2) encode enzymes or co-factors involved in the 5-lipoxygenase (5-LOX) cysteinyl leukotriene (cysLT) pathway that generates the cysLT metabolites: leukotriene C4 (LTC4), leukotriene D4 (LTD4) and leukotriene E4 (LTE4) (Fig 5e). Of note, the gene encoding the sixth enzyme in this pathway, leukotriene C4 synthase (LTC4S), also trended at greater than a two-fold enhancement in expression in the CBFβ^ΔLysM^ T1AECs, but lacked statistical significance.

Due to the current lack of mouse models that would allow us to selectively probe T1AECs *in vivo*, as well as the inability to sustain T1AECs in culture *ex vivo*, we utilized the recently created and characterized T1AEC cell line of C57Bl/6 origin, LET1 cells, to explore any link between AlvMΦ mediated resistance of T1AECs to IAV and the expression of these arachidonic acid pathway enzymes in T1AECs [28]. We first confirmed publish results that podoplanin positive LET1 cells can be infected with A/PR/8 NS1-GFP IAV as determined by GFP expression (Fig 5f) [28]. Consistent with our *in vivo* findings, primary AlvMΦs co-cultured directly with LET1 cells (or separated by a membrane barrier in transwell cultures) significantly reduced the infection of LET1 cells by IAV (Fig 5f). Co-culture of LET1 cells with splenic CD11c^+^ cells resulted in only a minimal increase in resistance of LET1 cells to infection, suggesting that the effect of the AlvMΦs on the LET1 cells was not due to a simple change in the MOI of the T1AECs in culture. We next utilized the LET1 and AlvMΦ co-culture system to determine if AlvMΦ mediated resistance to IAV was linked to the transcriptional inhibition of these arachidonic acid pathway enzymes in T1AECs. As Fig 5g demonstrates, consistent with our findings *in vivo*, the expression of genes encoding the arachidonic acid metabolism enzymes were reduced in infected LET1 cells co-cultured with AlvMΦs.

**Fig 6:**
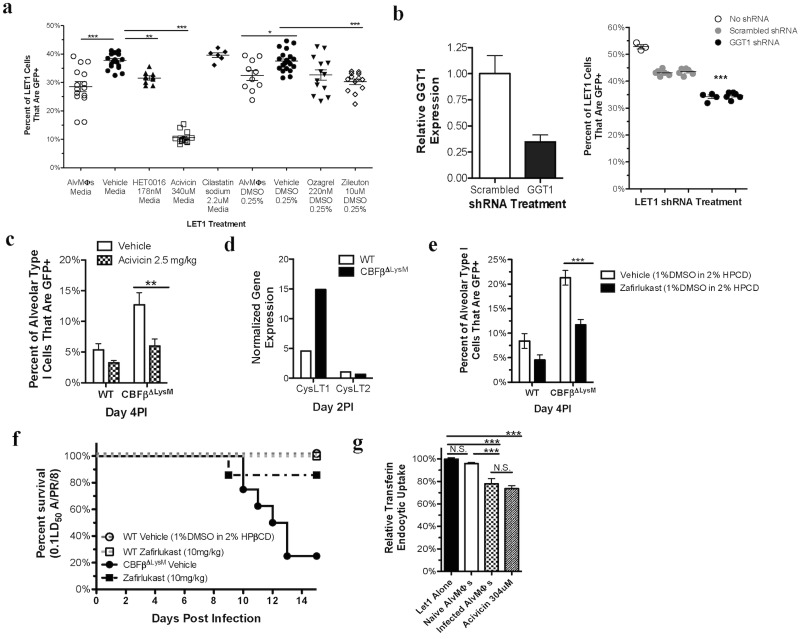
Inhibition of the 5-LOX pathway or blockade CysLT1 renders T1AECs resistant to IAV infection. a) Infectivity of LET1 cells infected with NS1-GFP A/PR/8 in the presence of the specified treatment. b) Lentivirus shRNA knockdown of GGT1 (left panel) and the impact LET1 cell infectivity (right). c) WT and CBFβ^ΔLysM^ mice were infected i.n. with NS1-GFP A/PR/8 and treated i.n. with 2.5mg/kg of Acivicin or vehicle at 5 and 29hours post infection. Infection of T1AECs (left) and conducting airway epithelial cells (right) was analyzed at day 4 PI. d) Expression of the CysLT1 and CysLT2 receptors in sorted T1AECs from WT and CBFβ^ΔLysM^ at day 2PI as determined by RNAseq. e) Day 4 T1AEC infectivity in WT and CBFβ^ΔLysM^ mice that were infected i.n. with NS1-GFP A/PR/8 and. f) CBFβ^ΔLysM^ mice that were infected i.n. with 0.1LD_50_ of A/PR/8 and treated i.p. with 10mg/kg of Zafirlukast or vehicle every 24 hours starting at 5 hours PI until day 3 PI. g) Relative fluorescence of pHrodo Red labeled transferrin that has been taken up via the endocytic route by LET1 cells in the presence of different treatments. For *in vitro* analyses, data were pooled from, or is representative of, a minimum of 3 experiments with each dot representing 2-pooled wells from a 24well plate. c and e) data was pooled 3 experiments for a total of 4–6 mice for each treatment and genotype. Error bars are standard error mean. Statistical analysis is a either a 2-way ANOVA, 1-way ANOVA, or a two-tailed non-paired students t test. * indicates P< .05, ** for P < .001 and *** for P < .001.

To determine if the activity of one or more of these arachidonic acid metabolism enzymes facilitates IAV infection of T1AECs, we examined the impact of small molecule inhibitors targeting these enzymes on the susceptibility of LET1 cells to IAV infection. Inhibition of thromboxane synthase enzymatic activity in LET1 cells with the inhibitor Ozagrel had minimal or no impact on IAV infection of the LET1 cells (Fig 6a). On the other hand, inhibition of enzymes along the cytochrome P450 F and A family pathway (by the small molecule inhibitor HET0016) resulted in a modest, statistically significant impact on IAV infection of LET1 cells (Fig 6a). Furthermore, inhibition of the activity of the 5-LOX pathway enzyme ALOX5 by Zileuton also produced a similar significant reduction in LET1 cell susceptibility to IAV infection (Fig 6a). We next examined the impact of inhibition of the activity the two enzymes downstream of ALOX5 in the cysLT pathway that were likewise upregulated in T1AECs from the AlvMΦ deficient mice. Inhibition of the activity of gamma glutamyl transferase-1 (GGT1) (which converts the metabolite LTC4 to LTD4) in LET1 cells alone with the drug Acivicin markedly reduced the susceptibility of LET1 cells to IAV infection. Conversely, inhibition of the enzyme DPEP2 (which converts LTD4 to the less biologically active metabolite LTE4) with the inhibitor Cilastatin had no effect on T1AEC susceptibility to infection (Fig 6a), suggesting that LTD4, but not LTE4, may regulated the susceptibility of T1AECs to IAV infection.

AlvMΦs, which render T1AECs resistant to IAV infection *in vivo* and *in vitro*, also reduced GGT1 expression *in vivo* and *in vitro* (Fig 5b and 5c). Inhibition of GGT1 enzymatic activity in T1AECs by Acivicin, likewise, decreased T1AEC susceptibility to IAV infection. Because Acivicin is not a selective GGT1 inhibitor, we next determined if knockdown of GGT1 expression in LET1 cells would also reduce their susceptibility to infection. As Fig 6b shows, compared to cells expressing control-scrambled shRNA, partial knockdown of GGT1 expression by GGT1 shRNA lentivirus treatment (Fig 6b left panel) also reduced the susceptibility of shRNA treated LET1 cells to IAV infection (Fig 6b right panel).

Although the interaction of AlvMΦs with LET1 cells *in vitro* recapitulated the effect of AlvMΦs on the susceptibility of T1AECs *in vivo*, this *in vitro* interaction likely does not reflect the full impact of AlvMΦs on T1AECs during IAV infection *in vivo*. Therefore, in order to determine if LTD4 production impacted the susceptibility of T1AECs *in vivo*, we evaluated the effect of Acivicin administration to WT and CBFβ^ΔLysM^ mice on the susceptibility of T1AEC. Acivicin was administration at 5 and 29 hours PI i.n. to inhibit early LTD4 production. These time points are prior to the large influx of CD45^+^ inflammatory immune cells into the lung, which can also be a significant source of cysLT metabolites [26]. *In vivo* Acivicin treatment markedly reduced the susceptibility of T1AEC from CBFβ^ΔLysM^ mice to IAV infection. Of note, Acivicin had only a minimal effect on the susceptibility of T1AECs from the AlvMΦ sufficient WT mice (Fig 6c). This latter finding suggests that the ability of Acivicin to reduce the susceptibility of T1AECs to infection is not due to nonspecific suppression of IAV infection.

The above evidence supported a link between the activity of the 5-LOX/cysLT metabolic pathway in T1AEC and their susceptibility to IAV infection. This is of interest as elevated levels of LTE4, the stable terminal metabolite of the cysLT pathway, has been reported to correlate with increased severity of IAV infection in both humans and mice [39]. LTD4 signals by engaging the cell surface Cysteinyl LT receptors (CysLT1 and/or CysLT2). The aforementioned RNAseq analysis revealed that at day 2 PI T1AECs express CysLT1 with twenty-two-fold higher FPKM (Fragments per Kilobase Mapped) than CysLT2 (Fig 6d). This is of particular interest as CysLT1 has higher affinity for LTD4 than CysLT2.

In order to explore the possibility that LTD4 engagement of CysLT1 on T1AECs was responsible for the enhanced susceptibility of T1AECs in the AlvMΦ deficient CBFβ^ΔLysM^ mice, we administered the CysLT1 antagonist Zafirlukast to these mice. As Fig 6e demonstrates, like Acivicin, i.p. administration of Zafirlukast on days 0–3 PI, markedly reduced T1AEC susceptibility to IAV infection in CBFβ^ΔLysM^ mice. Consistent with this data, Zafirlukast administration also rendered the treated CBFβ^ΔLysM^ mice resistant to lethal IAV infection (Fig 6f). These data are consistent with the concept that AlvMΦs act, at least in part, to suppress the susceptibility of T1AECs to infection by inhibiting cysLT pathway, possibly in T1AECs, which in-turn suppresses LTD4 signaling through CysLT1 on T1AEC.

The above findings suggest that engagement of CysLT1 on T1AECs enhances the susceptibility of these cells to infection by IAV. It has previously been reported that the initial enzyme in the Arachidonic Acid Metabolic Pathway, Phospholipase D, may facilitate IAV uptake [40]. Furthermore, key signaling molecules activated downstream of LTD4’s engagement of CysLT1 (ROCK1 and RAC) have been demonstrated to facilitate endocytic uptake of IAV [41, 42]. Therefore, we next sought to determine if AlvMΦs, which inhibit the arachidonic acid metabolism and cysLT pathway, also modulate endocytosis in T1AEC by evaluating receptor-dependent endocytic uptake of transferrin. As Fig 6g demonstrates, IAV-infected AlvMΦs, but not naïve AlvMΦs, decreased LET1 cell receptor-mediated clathrin-dependent endocytosis of transferrin that was labeled with the pH-sensitive dye pHrodo Red. Similarly, inhibition of GGT1 activity with Acivicin also decreased LET1 cell endocytic uptake of pHrodo Red labeled transferrin (Fig 6g). These findings are consistent with our data demonstrating AlvMΦ-mediated suppression of the cysLT pathway and protection of T1AECs from IAV infection *in vitro* and *in vivo*.

## Discussion

In this report we have evaluated the contribution of AlvMΦs to the host response in experimental IAV infection. We observed that mice with a genetic deficiency selectively in AlvMΦs (CBFβ^ΔLysM^ mice) are highly susceptible to IAV infection. This deficiency in AlvMΦs resulted in increased susceptibility of T1AECs to IAV infection that could be rescued by transferring WT AlvMΦs into the CBFβ^ΔLysM^ mice, suggesting that AlvMΦs are important mediators of T1AEC resistance to IAV infection. Along with increased numbers of infected T1AEC, the IAV infected AlvMΦ deficient mice also exhibit severely compromised pulmonary function and morphologic evidence of diffuse alveolar damage, which are compatible with severe lethal IAV pneumonia. While Type I and Type III IFNs are essential for respiratory epithelial cell resistance to IAV infection (including T1AEC), AlvMΦ-mediated protection of T1AECs was IFN-independent. Rather, our data strongly implicates that AlvMΦ-mediated transcriptional suppression of the cysLT pathway enzymes in T1AECs is one possible mechanism that mediates T1AEC resistance to IAV infection. Consistent with this resistance mechanism, inhibition of the cysLT pathway enzymes, in particular GGT1 reduced the susceptibility of T1AECs in CBFβ^ΔLysM^ mice to infection, as did antagonism of the cysLT receptor CysLT1.

Earlier reports evaluating the host response to IAV infection demonstrated a critical role for AlvMΦs in modulating the severity and outcome of IAV infection [9–13, 19]. While AlvMΦ phagocytosis of IAV particles, cellular debris and clearance of edema fluid undoubtedly contributes to AlvMΦ-mediated protection during IAV infection, a direct link between AlvMΦ function and the development of lethal pneumonia was not established in these reports. While it is currently not possible to directly assay cysLT metabolite production exclusively by T1AEC *in vivo* or *ex vivo*, to probe T1AEC CysLT receptor activity *in vivo* or *ex vivo*, or to disrupt this arachidonic acid metabolic pathway specifically in T1AECs *in vivo*, our results strongly suggest that there is a link between AlvMΦ-mediated resistance of T1AECs to IAV infection and AlvMΦ-mediated suppression of the expression of genes evolved in the cysLT pathway in T1AECs. This was evident both *in vivo*, where T1AEC from infected AlvMΦ deficient mice exhibited elevated expression of genes encoding cysLT pathway enzymes, as well as *in vitro*, where co-culture of T1AEC with AlvMΦs during IAV infection reduced both the expression of cysLT pathway genes and IAV infection. Additional support for a role of the cysLT pathway enzymes in regulating the efficiency of T1AEC IAV infection came from the effect of inhibiting 5-LOX (by the inhibitor Zileuton) and GGT1 (by the inhibitor Acivicin) specifically in LET1 cells. Even more compelling was the finding that treatment of infected CBFβ^ΔLysM^ mice and WT mice with Acivicin within the first two-days post infection significantly reduced the frequency of infected T1AEC in the AlvMΦ deficient CBFβ^ΔLysM^ mice, but had minimal effect on infection of T1AEC from infected WT mice. While our findings most likely reflect the impact of a quantitative deficiency in the number of AlvMΦs in the lungs of the CBFβ^ΔLysM^ mice, we cannot formally exclude the possibility that there is a subtle change in the function of the few residual AlvMΦs detected in the lungs. Likewise, we also cannot formally exclude that alterations in the function of other LysM expressing cell types lacking CBFβ could also affect the susceptibility of T1AECs to infection, and therefore the outcome of infection in the CBFβ^ΔLysM^ mice. However, findings on the effect of AlvMΦ transfer into CBFβ^ΔLysM^ mice, as well as the effect of acute depletion of AlvMΦ in the CD11c-DTR model, strongly implicate AlvMΦs as having a primary role and mediating resistance of T1AECs to infection.

The timing of AlvMΦ-mediated protection, along with the timing of cysLT gene activation and the brief, early timeframe in which Acivicin treatment worked have two important implications. First is that *in vivo* T1AECs are protected at a time point when AlvMΦs would be the predominant, if not exclusive, cell type in the alveoli to detect the infection and confer resistance to T1AEC. Therefore, it is formally possible that AlvMΦs are not the only CD45^+^ cell type capable of providing protection to T1AECs, and that it is their proximity to the AECs at this early time point that allows them to confer protection. Second, many of the CD45^+^ cell types that have been classically established as the cysLT producers have yet to substantially accumulate in the lungs or reach the airways by day 2 PI, which, once again, is when our data, particularly on Acivicin administration, strongly suggests that the cysLT metabolites are acting on the T1AECs to enhance their susceptibility to IAV infection. The finding that T1AECs may produce cysLTs is further supported by a growing body of literature demonstrating that, while initially *in vitro* data indicated that only myeloid cells express 5-LOX and therefore produce cysLTs [43], *in* vivo non-hematopoietic cell types as diverse as neurons, epithelial cells and endothelial cells are capable of expressing 5-LOX and producing cysLTs [44–50].

Methylation of the ALOX5 gene promoter has been reported in cell lines where the cysLT pathway is inactive. Therefore, methylation of ALOX5 has been suggested as the mechanism of gene silencing that accounts for the lack of 5-LOX expression and cysLT production by these cell types [48–50]. Since AlvMΦs prevent the upregulation of the cysLT pathway genes in T1AECs, AlvMΦs may confer resistance of T1AECs to infection by maintaining the ALOX5 promoter in a methylated state. As far as we are aware, there is minimal information concerning mechanisms to account for suppression, particularly transcriptional suppression of the cysLT pathway beyond methylation of the ALOX5 gene. We therefore attempted to identify the suppressive factor by screen the BAL fluid for inflammatory mediators that maybe differentially represented in the CBFβ^ΔLysM^ mice at day 2 PI when the 5-LOX and the cysLT pathway is suppressed by AlvMΦs. However, as noted above (Results), a 30-plex-cytokine/chemokine survey revealed no detectable differences in the inflammatory mediators present in the BAL at days 0 and 2 PI.

Activation of the cysLT pathway results in the synthesis of the cysLT metabolites LTC4, LTD4 and LTE4. A role for LTD4 engagement of CysLT1 on T1AECs was supported by the evidence that blockade of CysLT1 through Zafirlukast administration markedly reduced T1AEC susceptibility to IAV infection and prevented IAV associated mortality in CBFβ^ΔLysM^ mice. These findings raise the possibility that T1AEC production of the CysLT metabolite LTD4 may support cellular uptake or replication of IAV in T1AEC by signaling through cysLT receptors displayed by T1AEC.

Engagement of CysLT1, a G-protein coupled receptor, by LTD4 results in the mobilization of intracellular calcium. IAV has been reported to utilize calcium dependent activation of the ROCK-1 and RAC-1 signaling pathways in cells to facilitate IAV virion uptake and internalization [41, 42]. Constant with this work, we found that IAV-infected AlvMΦs were able to suppress LET1 cell endocytic uptake of transferrin. Signaling through CysLT receptors can also result in calcium-dependent activation of PI-3 kinase and CAM kinase in cells, which in turn enhances IAV gene expression in cells infected with certain IAV strains [41]. Thus the enhanced susceptibility of T1AEC to IAV infection in the absence of AlvMΦs may reflect both increased efficiency of virus uptake and the extent of virus replication as a result of CysLT1 engagement.

While we observed an increase in the frequency and absolute number of infected T1AEC in CBFβ^ΔLysM^ mice, overall lung virus titers in these animals only trended towards a slight enhancement and were only modestly elevated compared to lung virus titers in WT mice. This was in contrast to earlier reports in other mouse models [12] where an AlvMΦ deficiency resulted in elevated pulmonary virus titers. This discrepancy is perhaps not unexpected as the deficiency in AlvMΦs numbers is quantitative in our model, and T1AECs, while capable of productive IAV infection, are not as efficient in virion production as conducting airway epithelial cells [28]. Therefore, the 3-fold increase in infected T1AECs in the AlvMΦ deficient mice may not significantly impact the level of detectable virions in the infected lungs. We also demonstrate that the enhanced mortality of CBFβ^ΔLysM^ mice after IAV infection is likely not a direct result of virus mediated destruction of the increased number of IAV infected cells. Rather, our results suggest that the increased number of infected T1AEC rendered these cells more susceptible to adaptive-mediated elimination, and as a consequence, the development of lethal diffuse alveolar damage.

In conclusion, we have identified a novel role for AlvMΦs in modulating the severity of IAV infection by regulating the expression of the cysLT pathway in T1AECs and, as a consequence, the susceptibility of T1AECs to IAV infection. In addition to the mechanism proposed in this manuscript, AlvMΦs are likely to act though other IFN-dependent and—independent mechanism to limit IAV severity. However, our findings raise the possibility that therapeutic strategies to limit the susceptibility of T1AECs to infection, including blockade or antagonism of CysLT receptor signaling, early in infection could limit the development and severity of lower respiratory tract IAV infection.

## Materials and Methods

### Ethics Statement

This study was carried out in strict accordance with the Animal Welfare Act (Public Law 91–579) and the recommendations in the Guide for the Care and Use of Laboratory Animals of the National Institutes of Health (OLAW/NIH, 2002). All animal experiments were performed in accordance with protocols approved by the University of Virginia Animal Care and Use Committee (ACUC; Protocol Number 2230) [26].

### Mice and Infection

All mice were breed and housed in a pathogen-free environment and used at 7–14 weeks of age for all experiments. NS1-GFP virus was a generous gift from the Adolfo Garcia-Sastre laboratory. Influenza A viruses PR8 (H1N1) and NS1-GFP [27] were grown in the allantoic cavity of day 10 chicken embryos as described previously [26]. Mice were infected with 250 EID_50_ units of PR8 (0.1LD_50_), or 10^5^ EID_50_ NS1-GFP [26]. All infectious doses were administered i.n. in 50μL of serum-free Dulbecco’s Modified Eagle Medium (Invitrogen) following ketamine and xylazine anesthesia. For i.n. transfer of cells, 500,000 AlvMΦs were given in 50uL of serum-free Dulbecco’s Modified Eagle Medium (Invitrogen) following ketamine and xylazine anesthesia. Irradiation and bone marrow transplantation mice were irradiated with 9.5 Gy and, within 24hours, i.v. injected with RBC-lysed bone marrow cells (1–3 × 10^6^) [26]. For AlvMΦ depletion CD11c-DTr+ and CD11c-DTr- BALB/C littermates were given 40ng of DTx i.n. following ketamine and xylazine anesthesia. Acivicin was diluted in serum-free Dulbecco’s Modified Eagle Medium (Invitrogen) and 2.5mg/kg was given i.n. in 50uL following ketamine and xylazine anesthesia. 10mg/kg of Zafirlukast was given daily on days 0–3 PI by i.p. injection in 1mL of saline with 1%DMSO and 2% hydroxypropyl-β- cyclodextrin (HPCD).

### Sample Preparation

Mice were euthanized via cervical dislocation. Lungs were then perfused with PBS via the heart. Lungs were enzymatically digested with Type II collagenase (37°C for 30 minutes; Worthington) for analysis of hematopoietic cells. For epithelial cell analysis lungs were inflated and digested with Dispase 2 (37°C for 30 minutes; Invitrogen). Digestion was followed by passage through a steel mesh screen to remove tissue fragments. Red blood cells in the cell suspensions were lysed using ammonium chloride. Cells were enumerated using a hemocytometer. Cells were re-suspended in FACS buffer containing PBS, 2% FBS, 10mM EDTA, and 0.01% sodium azide for Ab staining or MACS buffer containing PBS, 2% FBS, and 10 mM EDTA.

### Bronchoalveolar Lavage Fluid (Cytokine, Viral Titer and Anti-Influenza IgG)

We obtained BAL fluid by flushing the airways three times with a single inoculum of 500uL sterile PBS introduced via a trachea incision. BAL fluid cytokine content was determined using the Luminex 100 IS system maintained by the UVA Flow Cytometry Core. The 30-factors assayed were: Eotaxin, GM-CSF, IFNg, IL-1a, IL-1b, IL-2, IL-4, IL-3, IL-5, IL-6, IL-7, IL-9, IL-10, IL12p40, IL-12-70, LIF, IL-13, IL-15, IL-17, IP-10, KC, MCP-1, MIP-1a, MIP-1b, M-CSF, MIP-2, MIG, RANTES, TNF. Viral titer was determined via endpoint dilution assay and expressed as tissue culture infectious dose_50_ (TCID_50_) units as previously described [26]. We incubated MDCK cells (ATCC collection) with tenfold dilutions of BAL fluid in serum-free trypsin supplemented DMEM culture medium. After 3–4 day incubation at 37°C in a humidified atmosphere of 5% CO_2_, culture supernatants were collected and mixed with a half- volume of 1% chicken red blood cells (University of Virginia Veterinary Facilities) to detect virus replication by hemagglutination. Detectable hemagglutination indicated virus replication was used as the calculate sample TCID50 values [26]. Influenza specific IgG antibodies in the airspaces were quantified by coating ELISA plates with A/PR/8 and incubating with tenfold dilutions of BAL fluid from influenza virus-infected mice. After washing, anti-mouse IgG was used to detect the amount of influenza specific IgG antibodies that where present in to BAL fluid at day 11 PI.

### Flow Cytometry Staining, Analysis, and Sorting

All FACS antibodies are purchased from BD Biosciences or eBioscience. The dilution of surface staining antibodies was 1 in 200 for 30 min at 4°C. After antibody staining, we examined cells using a six or eight-color FACS-Canto system (BD Biosciences) and the data were analyzed by FlowJo software (Treestar) and FMO or isotype controls were used to set gates. We characterized the epithelial cell types as follows: Conducting airways (CD45^-^ CD31^-^ T1α^-^ EpCAM^+^ MHCII^-^) and T1AECs (CD45^-^ CD31^-^ T1α^+^ EpCAM^+^). AlvMΦs were isolated from whole lungs by MACS enrichment for cells expressing either CD11c or Siglec F according to manufactures protocol generating around a 90% pure AlvMΦ population. T1AEC sorting was done using a modified protocol for sorting cells from culture for RNAseq analysis [51]. Briefly, T1AECs were stained and sorted directly into Trizol LS from whole lung suspensions in the presence of RiboLock using the Becton Dickinson Influx Cell Sorter and DEPC treated 1XPBS.

### Quantitative Reverse-Transcription PCR

We isolated RNA from the lungs via Triazol (Invitrogen) and treated it with DNase I (Invitrogen). We used random primers (Invitrogen) and Superscript II (Invitrogen) to synthesize first-strand complementary DNAs from equivalent amounts of RNA from each sample. We performed real-time RT-PCR in a 7000 Real-Time PCR System (Applied Biosystems) with SYBR Green PCR Master Mix (Applied Biosystems). Data were generated by the comparative threshold cycle (ΔCT) method by normalizing to HPRT [26]. Forward and reverse primers amplifying are as follows, respectively:

**M2**:5′GAGGTCGAAACG CCT 3′ & 5′CTGTTCCTTTCGATATTCTTCCC3′, **CYP4F18:**5′ AGAGCCTGGTGCGAACCTT 3′ & 5’ TGGAATATGCGGATGACTGG 3’, **CYP4F16:**5’GGAGTGGCTTCCTGGATTTT3’& 5’ATGCAGGGTCAACAATCCTC3’, **TBXAS1:**5’AGGCTTCTGAAAGAGGTGGACCT3′ & 5′TGAAATCACCATGTCCAGATAC3′, **ALOX5:**5′ATGCCCTCCTACACTGTCAC3′ & 5′CCACTCCATCCATCTATACT3′, **ALOX5ap:**5’CTCCCAGATAGCCGACAAAG3’ & 5’CAGAACTGCGTAGATGCGTA3’, **COTL1:**5’GATGAGGGCAAACTTGGATCT3’ & 5’GAGCAGATTACCAGCACTTCA3’, **GGGT1:** 5’AGGAGAGACGGTGACT3' & 5' GGCATAGGCAAACCGA3', **DPEP2:** 5’CTGACCTTTCTCTGCCACA3’ &5’GAATCTTCCTGATGACCTCCTG3’

### Evans Blue Dye

At the indicated day after infection with influenza, approx. 20mg/kg of Evans Blue dye in 500uL of 1X PBS was administered via the i.v. route. One hour later bronchoalveolar lavage fluid was obtained as described above. The absorbance of the dye at 620nm and 740nm was measured in BAL following removal of cells and debris and quantified with a standard curve obtained at the same time.

### Histology

Lungs were inflated with air using a sterile syringe and an intra-tracheal incision. The inflated lung was tied of and placed into Bouin’s Fix Solution for at least three days. Fixed lungs were taken to UVA’s Research Histology Core for Paraffin-embedding, slicing and Hematoxylin and Eosin staining.

### Measurement of Pulmonary Function

The MouseOx Pulse-oximeter (Starr Life Sciences, Oakmont PA) was used to measure blood oxygen saturation (SpO_2_). Prior to infection thigh hair of all mice was removed. Following ketamine and xylazine anesthesia, the thigh clamp was placed on the mouse and reading where taken on each mouse until it recovered from anesthesia. Oxygen saturation measurements were taken during recovery from anesthesia when oxygen saturation measurements had plateaued and only reading deemed successful by the software were used in our analysis.

### LET1 Cell Culture, Infection and Treatment

Let1 cells (a gift from Paul Thomas, St. Jude Hospital) were cultured and infected as previously described [28]. Briefly, 50,000 LET1 cells/ well were allowed adhere to wells of a 24 well tissue culture plate for 18hours in DMEM containing 10% FBS and antibiotics. Cell monolayers were then washed with OptiMEM to remove serum and non-adherent cells and then infected with A/PR/8 NS1-GFP in OptiMEM at an M.O.I. of ~100 to insure maximum infection of cells. Infection was carried out for 24hours, after which cells were liberated from the wells by manual manipulation. Two wells were pooled for each sample and live cells were analyzed by flow cytometry for T1α and GFP expression. For co-culture of Let1 cells with AlvMΦs, 100,000 AlvMΦs were added directly onto the LET1 cells or into transwell inserts at the time of LET1 plating and the same infection protocol mentioned above was followed. All drugs were introduced into the LET1 cell cultures at the time of infection at the specified concentrations, with or without DMSO, in OptiMEM. For stable knockdown of GGT1 in LET1 cells, lentiviral particles with control scrambled or GGT1 targeted shRNA were purchased from Santa Cruz Biotechnology (sc-35474-v) and used according to the manufactures protocol. Briefly, after an overnight incubation in 12 well plates, media containing Polybrene and 5 or 10uL of lentiviral particles was added. Cells were incubated overnight at 37°C and stable infection was selected for by maintaining the cells in media containing Puromycin dihydrochloride at 5ug/mL. For transferrin experiments, AlvMΦs were exposed to virus for 15 minutes at 4°and 20min at 37°. After virus was washed off the AlvMΦs were added to inserts and co-cultured with serum starved LET1 cells for one hour. pHrodo Red labeled transferrin (molecular probes) was then added to LET1 cells as per the manufacture’s protocol for 10 minutes at 16° followed by a 30 minute incubation at 37°. Fluorescence intensity was determined by flow cytometry.

### RNAseq

Cells were sorted as described above, stored at -80°C and shipped to BGI Americas in trizol. There, RNA was isolated, enriched by poly-A-selection, and amplified. Following this samples were barcoded and sequenced using a 101PE lane on a HiSeq 2000 sequencer by Illumina.

Data were processed with the Tuxedo Suite software package [52]. Paired-reads were aligned and mapped to the GRCm38 mouse genome assembly, followed by differential expression analysis. Gene expression pathway analysis was carried out using the DAVID bioinformatics database [37, 38]. GEO accession number GSE93085.

### Statistical Analyses

Data are means ± SEM. We used non-paired Student's t test, one-way ANOVA or two- way ANOVA for statistical analyses. We considered all P values >0.05 not to be significant.

## Supporting Information

S1 Fig Gating strategy for innate immune cells. a) CD45^+^ cells were gated into b) Eosinophils CD11c^-^ and Siglec F^+^ or AlvMΦs as CD11c^+^ and Siglec F^+^, which were further defined by CD11b expression. c) Siglec F^-^ cells were then further characterized as neutrophils by CD11b^+^ and Ly6G^+^, interstitial macrophages by CD11b^+^ and F4/80^+^, or as IMNCs as CD11b^+^, F4/80^-^ and Ly6G^-^ with the latter then further being further characterized by the Ly6C expression. d) CD45^+^ cells with limited FSC and SSC properties gated as CD11c^+^, MHCII^high^, and B220^-^ were identified as rDCs, which are either CD11b^+^ or CD103^+^.
(TIFF)

S2 Fig Characterization of CBFβ^ΔLysM^ mice. Naïve WT and CBFβ^ΔLysM^ mice a) BAL Cytospin and b) pulmonary histology images. Splenic c) macrophages, neutrophils, IMNCs and DCs were quantified in naïve WT and CBFβ^ΔLysM^ mice. Kinetic analysis of BAL infiltrating d) neutrophils and c) IMNCs in A/PR/8 infected WT and CBFβ^ΔLysM^ mice.
(TIFF)

S3 Fig Pulmonary epithelial cell gating strategy. a) Gating strategy of CD45^-^, CD31^-^ cells for identifying T1AECs (CD45^-^, CD31^-^, EpCAM^+^, T1alpha^+^), conducting airway cells (CD45^-^, CD31^-^, EpCAM^+^, T1alpha^-^ and MHCII^-^), and T2AECs (CD45^-^, CD31^-^EpCAM^+^, T1alpha^-^ and MHCII^+^) (top panel) with validation of MHCII as a marker for T2AECs (bottom panel). b) GFP expression in T1AECs after infection with the NS1-GFP reporter A/PR/8 strain. GFP positivity was determined using T1AECs infected with the WT A/PR/8 strain that does not have a GFP reporter. c) Percent of (left) and total numbers of (right) infected T2AECs at day 4 & 7 PI. d) NS1-GFP A/PR/8 infected WT mice received either control (IgG) or neutrophil depleting antibody (IA8) every 48hours by IP injection starting at day -1 PI. T1AEC infection was assessed on day 4 PI. For statistical analysis a two-tailed non-paired students t test (d) or 2-way ANOVA (c) was used where appropriate. * indicates P< .05, ** for P < .001 and *** for P < .001; NS is not significant.
(TIFF)
