# Supplementary figures and images for: Alveolar Macrophages Prevent Lethal Influenza Pneumonia By Inhibiting Infection Of Type-1 Alveolar Epithelial Cells
Source: PLoS Pathog. 2017 Jan 13;13(1):e1006140. doi: 10.1371/journal.ppat.1006140 (PMC5268648; doi:10.1371/journal.ppat.1006140)

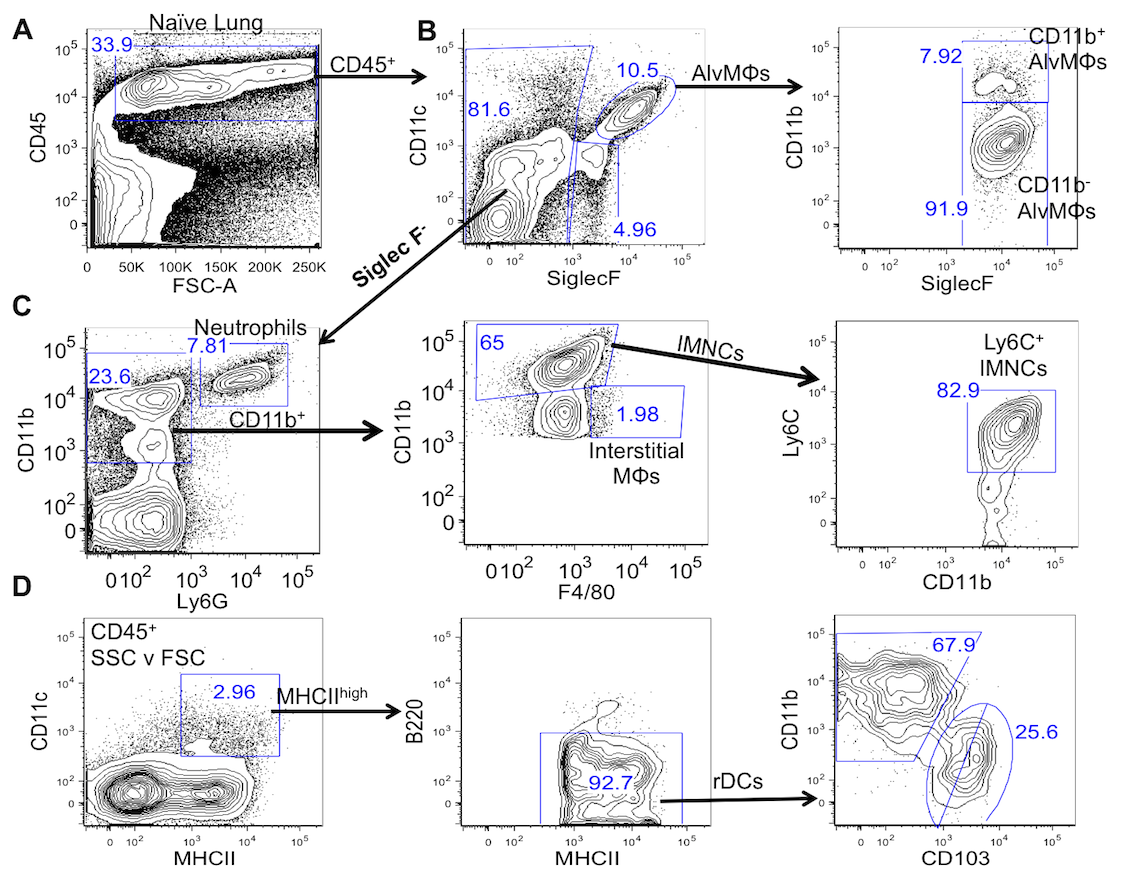

Supplement: S1 Fig — a) CD45+ cells were gated into b) Eosinophils CD11c- and Siglec F+ or AlvMΦs as CD11c+ and Siglec F+, which were further defined by CD11b expression. c) Siglec F- cells were then further characterized as neutrophils by CD11b+ and Ly6G+, interstitial macrophages by CD11b+ and F4/80+, or as IMNCs as CD11b+, F4/80- and Ly6G- with the latter then further being further characterized by the Ly6C expression. d) CD45+ cells with limited FSC and SSC properties gated as CD11c+, MHCIIhigh, and B220- were identified as rDCs, which are either CD11b+ or CD103+. (TIFF) [file ppat.1006140.s001.tiff]

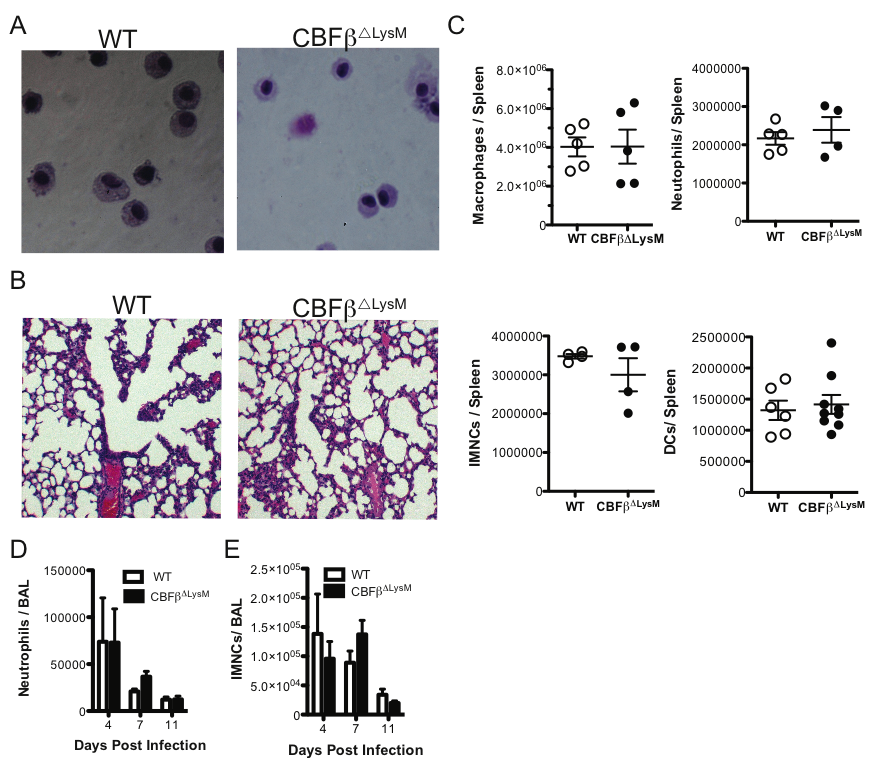

Supplement: S2 Fig — Naïve WT and CBFβΔLysM mice a) BAL Cytospin and b) pulmonary histology images. Splenic c) macrophages, neutrophils, IMNCs and DCs were quantified in naïve WT and CBFβΔLysM mice. Kinetic analysis of BAL infiltrating d) neutrophils and c) IMNCs in A/PR/8 infected WT and CBFβΔLysM mice. (TIFF) [file ppat.1006140.s002.tiff]

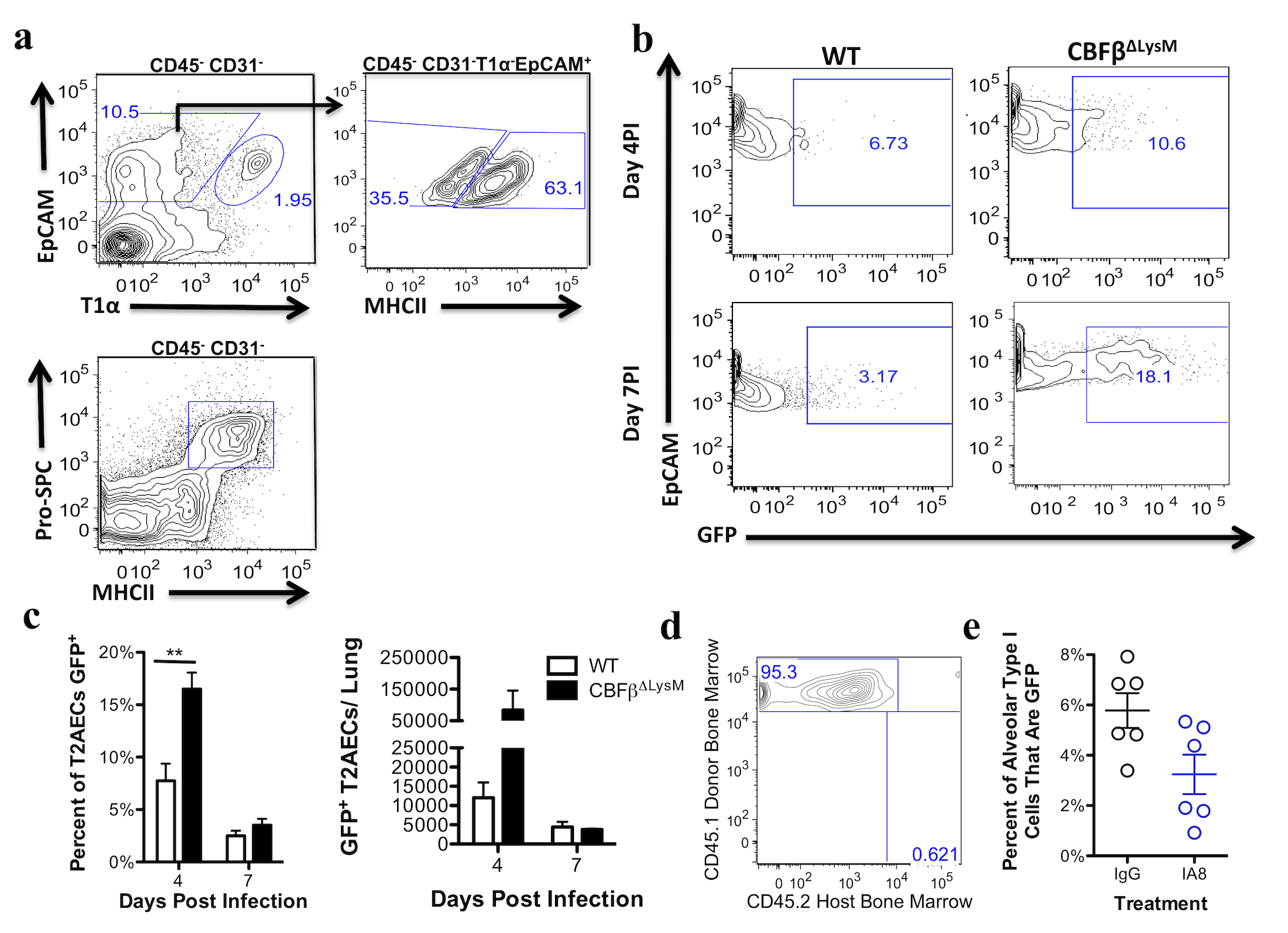

Supplement: S3 Fig — a) Gating strategy of CD45-, CD31- cells for identifying T1AECs (CD45-, CD31-, EpCAM+, T1alpha+), conducting airway cells (CD45-, CD31-, EpCAM+, T1alpha- and MHCII-), and T2AECs (CD45-, CD31-EpCAM+, T1alpha- and MHCII+) (top panel) with validation of MHCII as a marker for T2AECs (bottom panel). b) GFP expression in T1AECs after infection with the NS1-GFP reporter A/PR/8 strain. GFP positivity was determined using T1AECs infected with the WT A/PR/8 strain that does not have a GFP reporter. c) Percent of (left) and total numbers of (right) infected T2AECs at day 4 & 7 PI. d) NS1-GFP A/PR/8 infected WT mice received either control (IgG) or neutrophil depleting antibody (IA8) every 48hours by IP injection starting at day -1 PI. T1AEC infection was assessed on day 4 PI. For statistical analysis a two-tailed non-paired students t test (d) or 2-way ANOVA (c) was used where appropriate. * indicates P< .05, ** for P < .001 and *** for P < .001; NS is not significant. (TIFF) [file ppat.1006140.s003.tiff]
